# Chloroform and the Queen

**Published:** 1853-07

**Authors:** 


### CHLOROFORM AND THE QUEEN.

We stated in our last number upon what we deemed good authority, that, in her late accouchement, Queen Victoria was placed under the influence of chloroform. The London Lancet for May, referring to the report, which is given in the Association Medical Journal, has the following paragraph on the subject:

"On inquiry we were not at all surprised to learn that in her late confinement the Queen was not rendered insensible by chloroform, or by any other anæsthetic agent. Probably some officious meddlers about the court so far overruled her Majesty's responsible professional advisers as to lead to the pretence of administering chloroform."

The question is one of slight importance, but the denial of the Lancet does not appear to us to amount to anything. It supposes there might have been a "pretence of administering" the anæsthetic. We cannot help thinking it singular if the Queen and her "responsible professional advisers" were agreed to enact such a farce on an occasion of the kind.

We believe the first accident from the administration of chloroform in midwifery has yet to be recorded. The fatal cases in dentistry and surgery are, unhappily, but too numerous and too well attested. Why, in all the multiplied instances of its administration to parturient females, in natural as well as in difficult labor, not one casualty has occurred, while so many patients have died in the dentist's chair or on the surgeon's table, may be impossible to explain, but it is certainly a curious and interesting fact. It would seem to denote that it is in the practice of the obstetrician especially, that chloroform has its appropriate place and possesses its highest value. The pain of a woman in travail, we suspect, is as acute as any inflicted by the surgeon's knife, and if anæsthesia may be purchased on terms which seem so safe, we confess we are in favor of giving her the benefit of it, even in cases of natural labor. If our professional experience should change, and deaths begin to occur in midwifery as they have done in surgery, then we should certainly hesitate; but let us hope that we are not destined to meet with any such reverse.

